# Changes in dietary habits and weight status during the COVID-19 pandemic and its association with socioeconomic status among Iranians adults

**DOI:** 10.3389/fpubh.2022.1080589

**Published:** 2023-01-12

**Authors:** Maryam Maharat, Seyedeh Forough Sajjadi, Seyedeh Parisa Moosavian

**Affiliations:** Department of Community Nutrition, Vice-Chancellery for Health, Shiraz University of Medical Sciences, Shiraz, Iran

**Keywords:** COVID-19, dietary habits, weight, dietary supplements, socioeconomic status

## Abstract

**Background:**

COVID-19 pandemic has impacted human health through sudden lifestyle changes, including isolation at home, and social distancing. Therefore, the current study aimed to investigate the effect of the COVID-19 pandemic on eating habits, weight status, and their associations with socioeconomic status.

**Methods:**

This cross-sectional study was conducted using an online structured questionnaire that inquired demographic, anthropometric (reported weight and height); dietary habits (weekly intake of certain foods); and dietary supplement intake information.

**Results:**

A total of 1,187 participants completed the questionnaire, and after validation of the data, 1,106 respondents were included in the study, with a mean age of 34.5 ± 9.4 years. Our findings showed that the body mass index (BMI) of the participants significantly increased during COVID-19 (*P* < 0.001). Also, there were significant changes in the intake of a variety of food and beverage during the COVID-19, including less consumption of milk, yogurt, red meat, fish, canned fish, homemade fast foods, take out fast foods, carbonated drinks, and more consumption of whole bread, legumes (chickpeas, lentil, peas, kidney beans, black beans, pinto beans, and navy beans), soy bean, nuts, seeds, high vitamin C vegetables, high vitamin C fruits, green-yellow fruits and vegetables, onion/garlic, dried fruits, natural fruit juices, and water (*P* < 0.001; for all). It is informed that individuals consumed more vitamin and mineral supplements (*P* < 0.001). Also, before and during COVID-19 pandemic weekly intakes of dairy, red meat, poultry, high vitamin C fruits, and whole bread were positively associated with socioeconomic status (*P* < 0.001).

**Conclusion:**

Overall, this study indicates changes in body weight, dietary habits and supplement intake during the pandemic. Therefore, the findings of this study are valuable for, health professionals and politicians to better public health practice and policy making.

## Introduction

COVID-19 has dramatically expanded across the world since its first detection in Wuhan, China. The virus has reached nearly every country worldwide in < 6 months (1). Over half of the world's population is, or has been, under some form of social distancing or lockdown in an attempt to contain the health crisis. This has led to a deep alteration of the usual patterns of daily living, such as closure of businesses and a shift to a “working from home” business model (2), changes in social dynamics (3), reduced physical activity and increasing sedentary behavior (4). These societal changes have also led to alterations in individual's food practices (2). In addition, COVID-19 pandemic resulted in increased stress, anxiety or depression induced by the quarantine and disruption of daily routine, along with fear of infection (5, 6). Mental health impact hunger, food choices, and the desire to eat and food choices (7, 8). It has been reported that anxiety is associated with more consumption of high calorie, high fat and high sugar foods during COVID-19 pandemic (9–11).

COVID-19 pandemic may have both direct and indirect effects on food security and nutrition (12).

It is suggested that home confinement due to the COVID-19 may have led to better overall diet quality through more frequency of cooking and eating at home (13–15). However, this has not been a consistent finding. On the other hand, limited access to grocery shopping and panic buying during lockdown may reduce the consumption of fresh foods, especially fruit, and vegetables, in favor of unhealthy foods with longer shelf lives (16, 17). Also, lockdown indirectly decrease the financial capacity to purchase foods due to loss of work, even more so among more vulnerable populations, which leading to worse dietary habits and an overall diet of lesser quality (13, 18).

On the other hand, several researchers across the worldwide have observed weight gain during the COVID-19 pandemic due to poor food choices, physical inactivity, and social isolation (19–21). For example, the weight gain in Italy during the pandemic ranged between 1.5 and 3 kg (21). Also, an average of 0.62 kg weight increase had been reported in the United States (22).

Poor dietary habits along with an unhealthy lifestyle, can cause serious health problems. Therefore, in this critical period optimizing nutrition is essential (23, 24). Having knowledge about individuals' dietary habits may help prevent chronic conditions and their associated risks (24). In addition, both health professionals and governments use this data for public health practice, economic analysis and policy setting (24).

Iran is a middle-income country located in the Middle East. In this country, food security and nutrition situation varies by geography and demography. Unfortunately, Iranian households prioritize abdominal satiety over the consumption of nutritious foods. Overall, even before the COVID 19 pandemic took hold, the consumption of milk and dairy products, eggs, vegetables, and fruits by Iranians is low (25). Food price is the main factor in influencing people's food choices. The COVID 19 pandemic can change the quality of diet due to its impact on social and economic conditions (26). Therefore, the primary aim of this study was to investigate the effects of the COVID-19 pandemic on dietary intake among Iranian adults. The second is to examine the association between socioeconomic status and food intake before and during COVID-19 pandemic.

## Methods

### Study design

This cross-sectional study was conducted among Iranian adults during the COVID-19 out-break.

The inclusion criteria to participate in the study was age>18 years. We collected data using an online platform, accessible through any device with an Internet connection. The link for the e-form was forwarded throughout social media platforms (WhatsApp, and Instagram). Participants were asked to share the survey link with their family and friends. It was facilitated the wide dissemination of the survey questionnaire during the pandemic. This method provides a statistical collective whose population parameters cannot be controlled, as it is the case for probabilistic sampling. A brief description of the study and its intent was provided at the start of the survey. The study was anonymous, and participation was voluntary.

The sample size was estimated using Gpower software (α = 0.05, β = 0.2) according to the pervious study (27). Therefore, the minimum sample size required for this study is 515 respondents. A total of 1,187 participants responded to this survey; however, findings in the present study were based on the responses from 1,106 participants, after excluding participants who did not complete the questionnaire appropriately. The study protocol was approved by the Research Ethics Committee of Shiraz University of Medical sciences (IR. SUMS.REC.1401.344). This Web-based surveys reported according to the CHERRIES guidelines (28).

### Questionnaire

Data were collected through a digital questionnaire consisted of 4 sections. The first part gathers information about socio-demographic characteristics, including age, gender, education, physical activity. Socioeconomic status (SES) was defined based on scoring of the education, income, asset and wealth of their household (homeownership, personal vehicle, washing machine, LCD/LED TV, dishwasher, laptop/ computer, refrigerator, and microwave) variables. The questions and the assigned scores were as follows: education (lower than 12-year formal education = 1, 12-year formal education = 2, 12–16 year formal education =3, more than 16-year formal education = 4), income (lower than 100 United States dollar (USD) = 1, 100 to less than 200 USD = 2, 200 to less than 300 USD = 3, 300 USD and more = 4), asset and wealth (Having 3 items or less = 1, 4–6 items = 2, 7 items and more = 3). Then, participants were classified based on tertiles of SES score to low, middle, and high SES. Self-reported weight and height before and during the pandemic were obtained. Body mass index (BMI) was then calculated by dividing weight to high squared (m^2^). BMI status was classified based on the WHO categories (29) as follows: underweight (BMI < 18.5), normal weight (BMI between 18.5 and 24.9), overweight (BMI between 25 and 29.9) and obese (BMI ≥ 30). Physical activity level was estimated using the short-form of the International Physical Activity Questionnaire (SF-IPAQ). The SF-IPAQ questionnaire had been validated in Iran and the correlation coefficient for reliability was 0.7 (30). This questionnaire is composed of seven questions about physical activity in a typical week. The physical activity of the participants was calculated as metabolic equivalents (MET)-minutes/week. According to the guidelines for data processing and analysis of the IPAQ, participants were stratified into three categories [low (≤ 600 MET-minutes/week), moderate (600 to < 1,200 MET-min/week), and high levels (≥1,200 MET-min/week) of physical activity] (31). The second section asked about the participants' health status, such as chronic diseases (diseases with proven diagnosis), and whether they were previously infected with COVID-19. Based on the UK NHS report (32), if respondents had any of 10 medical conditions (e.g., diabetes, weakened immune system, chronic kidney disease…), they were considered as “high risk” for COVID-19. The third part of the questionnaire asked about the participants' dietary habits on before the COVID-19 outbreak and during the pandemic. A semi-quantitative food frequency questionnaire (SFFQ), which was validated by Keshteli et al. (33), was used to evaluate the eating behavior. The correlation coefficient for reliability was 0.77. Since this study was performed during the pandemic, we slightly shortened the questionnaire to prevent the adverse effects of the length of the questionnaire on the response rate. Intakes of each food item were recorded based on servings per week. The final part obtains data about nutritional supplement consumption.

### Statistical analysis

Data analysis was conducted using IBM SPSS version 26.0. Data are represented as number and percentage for quantitative variables, or median and interquartile range for quantitative data. Normality distribution of variables was evaluated using Shapiro–Wilk test. The Chi-square test was used to determine whether categorical variables differed. Mann–Whitney *U* and Kruskal–Wallis tests were performed to compare continuous variables. *P* < 0.05 is considered statistically significant.

## Results

A total of 1,187 participants completed the questionnaire, and, after validation of the data, 1,106 respondents have been included in the study, with a mean age of 34.5 ± 9.4 years. The socio-demographic characteristics of the study participants are indicated in Table 1. Most of the participants were female (85.2%). Statistically, the largest group was people aged 31–50 years. Moreover, they mainly lived in urban (76.2%). Most subjects had 12 years of education (45.1%), low physical activity (40.6%), and low SES (61%). In terms of marital status, 79.9% of participants were married. Furthermore, 32.8% of respondents had a high risk for COVID-19, and 32.1% had been diagnosed COVID-19. Hypothyroidism is the most prevalent diseases reported (10.3%).

**Table 1:** Socio-demographic characteristics of the participants who filled out the questionnaire.

**Variable**	***N* = 1,106**
**Gender**	
Female	943 (85.2)
Male	163 (14.7)
**Age (years)**	
18–30	354 (32)
31–50	704 (63.6)
51–60	48 (4.3)
**Place of living**	
Urban	843 (76.2)
Rural	263 (23.7)
**Education level (years)**	
< 12	189 (17)
12	418 (37.7)
12–16	399 (36.1)
>16	100 (9)
**Monthly income (USD)**	
< 100	475 (42.9)
100 < 200	415 (37.5)
200 < 300	162 (14.6)
≥300	54 (4.9)
**SES**	
Low	681 (61.5)
Medium	376 (34)
High	15 (1.3)
**Current marital status**	
Married (not separated)	862 (77.9)
Widowed or divorced	18 (1.6)
Single	226 (20.4)
**Physical activity**	
Low	450 (40.6)
Moderate	263 (23.7)
High	87 (7.8)
**Smoking habit**	
Yes	69 (6.2)
No	1,037 (93.7)
**Diagnosed COVID**	
Yes	356 (32.1)
No	750 (67.8)
**At risk medical group for COVID**	
Yes	363 (32.8)
No	743 (67.1)

SES, Socioeconomic status. Values are expressed as number (percentage).

1 US$ = 350,000 Rials.

As shown in Table 2, the BMI of the participants significantly increased during COVID-19 (*P* < 0.001). In comparison to the before COVID-19 pandemic, the number of the subject with overweight, and obesity increased significantly (*P* < 0.001).

**Table 2:** Weight status of participants before and during COVID.

	**Before COVID-19 (mean ±SD)**	**During COVID-19 (mean ±SD)**	***P***
Weight (kg)	68.18 ± 15	68.7 ± 14.59	< 0.001
BMI (Kg/m^2^)	25.8 **±** 5.7	26 **±** 6.57	< 0.001
**Class of BMI**	***N*** **(%)**	***N*** **(%)**	
Under weight	66 (6)	56 (5.1)	< 0.001
Normal	444 (40.1)	428 (38.7)	
Over weight	417 (37.7)	434 (39.2)	
Obese	179 (16.2)	188 (17)	

BMI, body mass index.

Values are expressed as mean ± standard deviation or number (percentage). P resulted from Mann–Whitney U test, and chi-squared test.

Comparison of dietary intake patterns before and during COVID-19 pandemic are presented in Table 3. During the COVID-19 pandemic, consumption of whole bread, legumes, soybean, nuts, seeds, high vitamin C vegetables, high vitamin C fruits, green-yellow fruits and vegetables, onion/garlic, dried fruits, natural fruit juices, and water increased significantly (*P* < 0.001). However, significant decrease were observed in the intake of milk, yogurt, red meat, fish, canned fish, homemade fast foods, take-out fast foods, and carbonated drinks (*P* < 0.001).

**Table 3:** Dietary intake patterns before and during COVID-19 pandemic.

**Food items**	**Before COVID-19**		**During COVID-19**		***P***
	**Mean ±SD**	**Median (IQR)**	**Mean ±SD**	**Median (IQR)**	
Whole bread (serving/week)	7.3 ± 10.39	4 (1–9)	7.49 ± 10.47	4 (1–9)	< 0.001
Legumes and beans (food spoon/week)	9.51 ± 9.26	6 (3–10)	9.89 ± 10.1	6 (3–12)	< 0.001
Soy bean (food spoon/week)	3.47 ± 5.1	2 (0–5)	3.54 ± 5.9	2 (0–5)	< 0.001
Nuts (number/week)	9.54 ± 16.39	5 (1–10)	9.85 ± 15.9	5 (1–10)	< 0.001
Seeds (food spoon/week)	4.37 ± 6.49	3 (1–5)	4.57 ± 7.55	3 (1–5)	< 0.001
Milk (serving/week)	1.85 ± 2.5	1(0–3)	1.65 ± 2.33	1 (0–2)	< 0.001
Yogurt (serving/week)	2.79 ± 2.23	2 (1–4)	2.62 ± 2.25	2 (1–4)	< 0.001
Cheese (serving/week)	3 ± 2.6	3 (2–5)	3 ± 2.5	3 (2–5)	0.060
Red meat (serving/week)	4.41 ± 4.46	3 (2–5)	4.31 ± 4.72	3 (2–5)	0.028
Poultry (serving/week)	4.6 ± 5.1	3 (2–5)	4.5 ± 4.9	3 (2–5)	0.052
Fish (serving/week)	1.42 ± 2.65	1 (0–2)	1.32 ± 2.6	1 (0–2)	< 0.001
Canned fish (serving/week)	0.72 ± 1.92	0 (0–1)	0.59 ± 1.76	0 (0–0)	< 0.001
Egg (number/week)	3.89 ± 2.69	3 (2–5)	3.97 ± 2.97	3 (2–5)	0.051
Homemade fast foods (serving/month)	1.94 ± 2.3	1 (1–2)	1.69 ± 2.29	1(0–2)	< 0.001
Take out fast foods (serving/month)	1.25 ± 2.12	1 (0–2)	0.85 ± 1.83	0 (0–1)	< 0.001
High vitamin C vegetables (serving/week)	3.95 ± 3.53	3 (2–5)	4.18 ± 4.06	3 (2–5)	< 0.001
High vitamin C fruits (serving /week)	5.3 ± 4.2	5 (2–7)	5.56 ± 4.9	5 (2–7)	< 0.001
Green, yellow fruits and vegetables (serving/week)	3.48 ± 3.22	3 (1–5)	3.18 ± 4	3 (1–5)	< 0.001
Onion/garlic (serving/week)	4.12 ± 3.96	3 (2–5)	4.73 ± 5	4 (2–6)	< 0.001
Dried fruits (number/week)	3.1 ± 5.6	1 (0–4)	3.28 ± 6	1 (0–4)	0.01
Natural fruit juices (glass/week)	1.19 ± 1.9	0 (0–2)	1.58 ± 2.34	1 (0–2)	< 0.001
Commercial fruit juices (glass/week)	0.92 ± 1.69	0 (0–1)	0.89 ± 1.7	0 (0–1)	0.152
Carbonated drinks (glass/week)	1.9 ± 2.3	1 (0–3)	1.69 ± 2.4	1(0–2)	< 0.001
Water (glass/day)	5.52 ± 4.22	5 (3–7)	6.46 ± 4.5	6 (4–8)	< 0.001

Values are expressed as mean ± standard deviation and median (interquartile range). P resulted from Mann–Whitney U test.

Dietary supplement intake before and during the COVID-19 pandemic is reported in Table 4. Vitamin C, zinc, multivitamin, Calcium + Vitamin D consumption increased significantly during the COVID-19 pandemic (*P* < 0.001). Moreover, compared to the before COVID-19 pandemic, the number of subjects who intake vitamin D increased notably (*P* < 0.001).

**Table 4:** Dietary supplement intake before and during COVID-19 pandemic.

**Supplement**	**Before COVID-19**		**During COVID-19**		***P***
	**Mean ±SD**	**Median (IQR)**	**Mean ±SD**	**Median (IQR)**	
Vitamin C (number/week)	1.67 ± 4.3	0 (0–1)	3.03 ± 6	0 (0–3)	< 0.001
Zinc (number/month)	1.49 ± 4.2	0 (0–0)	2.55 ± 6.8	0 (0–1)	< 0.001
Calcium (number/week)	0.56 ± 2.64	0 (0–0)	0.61 ± 2.43	0 (0–0)	0.09
Calcium + vitamin D (number/month)	1.05 ± 2	0 (0–1)	1 ± 3	1 (0–1)	< 0.001
Multivitamin (number/month)	2.31 ± 6.74	0 (0–2)	5.40 ± 10.08	1 (0–5)	< 0.001
**Vitamin D**	**N (%)**		***N*** **(%)**		
Use	690 (62.3)		745 (67.3)		< 0.001
Non-use	416 (37.6)		361 (32.6)		

Values are expressed as mean ± standard deviation or number (percentage). P resulted from Mann–Whitney U test and median (interquartile range), and chi-squared test.

Comparison of food intake in each socioeconomic status before and during COVID-19 pandemic are shown in Table 5. Before and during the COVID-19 pandemic, weekly intakes of dairy, red meat, poultry, high vitamin C fruits, and whole bread were positively associated with socioeconomic status (*P* < 0.001). However, consumption of egg was higher in respondents with low socioeconomic status than other before and during COVID-19 pandemic (*P* < 0.001). There were no significant differences in the intake of high vitamin C vegetables between groups.

**Table 5:** Food intake in each socioeconomic status before and during COVID-19 pandemic.

**Food (item)**	**Socioeconomic status**			
	**Low**	**Medium**	**High**	*P* ^a^
**Dairy (serving/week)**				
Before	7.8 ± 4.85	8.36 ± 5	10.67 ± 5.37	< 0.001
During	7.35 ± 5	7.81 ± 4.84	10.16 ± 5.25	< 0.001
*P* ^b^	< 0.001	< 0.001	0.523	
**Red meat (serving/week)**				
Before	4 ± 4.48	5 ± 4.48	5.47 ± 3.16	< 0.001
During	3.84 ± 4.78	4.99 ± 4.76	5.59 ± 3.14	< 0.001
*P* ^b^	< 0.001	0.954	0.472	
**Poultry (serving/week)**				
Before	4.16 ± 5	5.23 ± 5.13	5 ± 3.98	< 0.001
During	3.99 ± 5.18	4.97 ± 4.76	5.38 ± 4	< 0.001
*P* ^b^	< 0.001	0.05	0.179	
**Egg (number/week)**				
Before	4.01 ± 2.92	3.76 ± 2.31	3.92 ± 2	< 0.001
During	4.07 ± 3.16	3.80 ± 2.68	3.78 ± 2	< 0.001
*P* ^b^	0.238	0.851	0.300	
**High vitamin C vegetables (serving/week)**				
Before	3.86 ± 3.43	4.14 ± 3.84	3.67 ± 2.1	0.424
During	4 ± 4.23	4.38 ± 3.88	3.98 ± 2.7	0.203
*P* ^b^	0.007	< 0.001	0.210	
**High vitamin C fruits (serving/week)**				
Before	4.9 ± 4.12	5.86 ± 4.34	6.63 ± 4.11	< 0.001
During	4.97 ± 4.5	6.41 ± 5.48	7.08 ± 4.25	< 0.001
*P* ^b^	0.248	< 0.001	0.375	
**Whole bread (serving/week)**				
Before	6.49 ± 9.39	8.43 ± 11.76	9.96 ± 10.36	< 0.001
During	6.56 ± 9.61	8.5 ± 11.49	10.98 ± 12.3	< 0.001
*P* ^b^	0.051	< 0.001	0.287	

Values are expressed as mean ± standard deviation.

^*a*^P resulted from Kruskal–Wallis test.

^*b*^P obtained from Mann–Whitney U test.

Within-group analyses indicated that dairy, red meat, and poultry intake in subjects with low socioeconomic status significantly decreased (*P* < 0.001), and consumption of high vitamin C vegetables increased (*P* = 0.007) during COVID-19 out-break compared to the before pandemic.

Also, in subjects with medium socioeconomic status, mean intake of dairy significantly decreased (*P* < 0.001), however, high vitamin C vegetables, high vitamin C fruits, and whole bread consumption increased (*P* < 0.001) during COVID-19 out-break. No significant differences were identified in participants with high socioeconomic status in terms of food intake before and during COVID-19 pandemic.

## Discussion

The main goal of the present study was to evaluate how Iranian participants' dietary habits changed during COVID-19 pandemic. Our findings indicated that COVID-19 had a negative effect on BMI. In line with our study, previous findings reported that weight was increased during the COVID-19 (19, 20, 22, 34–38). Also Zhu et al. performed a cross-sectional study using an online questionnaires among 889 residents of Jiangsu and other provinces of China aged between 16 and 70 years and found an average gain weight of 0.5 kg during the pandemic (39), which was similar to the current study. In another online cross-sectional survey among 1,200 participants in USA, 22% of the sample stated they gained 5–10 pounds during the COVID-19 pandemic (19). Several factors may have effect on weight gain and obesity during the COVID-19 out-break, including sedentary behaviors, physical inactivity, and screen time (36, 40–42). Also, access to physical activity resources, such as sports clubs was limited due to the quarantine. An online longitudinal study showed that time of watching TV significantly increased in French-speaking countries (i.e., Belgium, France, and Switzerland) during the pandemic (43). Moreover, unhealthy dietary habits including, overconsumption, and high intake of canned food could be another factor related to weight gain during the pandemic (19, 34, 41, 44). An online cross-sectional survey conducted among individuals older than 18 years in Spain and reported that higher odds of weight gain were associated with increased consumption of sugary drinks, homemade pastries and fried food, eating more than usual, and increased snacking during the pandemic (44).

In addition, we informed significant changes in the intake of a variety of food and beverage during COVID-19, including less consumption of milk, yogurt, red meat, fish, canned fish, homemade fast foods (pizza, chicken Burger, hamburger, Cheeseburger, and so on) take out fast foods, carbonated drinks, and more consumption of whole bread, legumes, soy bean, nuts, seeds, high vitamin C vegetables, high vitamin C fruits, green-yellow fruits and vegetables, onion/garlic, dried fruits, natural fruit juices, and water. In line with our results, previous studies reported that milk, and yogurt intake significantly decreased during the pandemic (27, 45). Also, Jia et al. conducted a cross-sectional study using an online questionnaires among 10,082 chines adults and identified a significant decrease in the intake of red meat (45). With regard to dietary fish, Chinese individuals reduced their consumption of fish during the COVID-19 out-break (46). Also, another study which conducted among 1,553 Iranian adults using an online questionnaires reported that both fresh and canned fish intake significantly decreased in the outbreak period (27). The reduced consumption of dairy products, red meat, fresh and canned fish during the pandemic might be due to the lockdown/home confinement at this time (47).

The results of this study indicated a significant decrease in the intake of homemade fast foods, take out-fast foods, and carbonated drinks. A recent systematic review of the 32 studies conducted by Bakaloudi et al. observed a downward trend in fast-food consumption (48). Kriaucioniene et al. in an online cross-sectional survey among 2,447 individuals older than 18 years identified that intake of carbonated or sugary drinks, fast food and commercial pastries decreased in Spain during the COVID-19 out-break (44). It seems possible that long time staying at home and increased free time resulting from quarantine made individuals to spend more time in cooking (34, 49, 50). Another reason could be the tendency to eat healthier foods in reaction to COVID-19 out-break (7). Finally, it could be the outcome of the fear from the transmission of COVID-19 disease *via* unhygienic practices at restaurants or delivery services (45).

We found that before and during pandemic weekly intakes of dairy, red meat, poultry, high vitamin C fruits, and whole bread were positively associated with socioeconomic status. In contrast, egg intake was higher in respondents with low socioeconomic status than other before and during COVID-19 pandemic. In the present study, higher socioeconomic status is associated with higher educational level and income. A growing body of research found an increasing trend toward a better quality diet with the increase in socioeconomic status (51). Gómez et al. examined the effect of socioeconomic status (SES) on diet quality using data from the “Latin American Health and Nutrition Study (ELANS),” a multi-country (Argentina, Brazil, Chile, Colombia, Costa Rica, Ecuador, Peru and Venezuela), population-based study of 9,218 participants, and found that participants from the low SES consumed less fruits, vegetables, whole grains, fiber and fish and seafood and more legumes than those in the high SES. Also, the diet quality level, assessed by DQS (dietary quality score), DDS (dietary diversity score) and NAR (nutrients adequacy ratio) mean, increased with SES (52). López-Olmedo et al. analyzed data from adults participating in the subsample with dietary information from the Mexican National Health and Nutrition Survey 2012 (*n* = 2,400), and they found that a lower educational level and lower assets index were positively associated with higher Mexican Diet Quality Index scores (53). In agreement with our results, a positive association between belonging to a higher level of SES and consumption of meats, dairy, and fruits were observed in other studies (54, 55). However, the higher consumption of eggs in people with low SES might be due to the lower price of it than other animal proteins.

On the other hand, decrement of dairy, red meat, and poultry intake, and increment of high vitamin C vegetables in people with low SES during pandemic might be due to replacing expensive food with high vitamin C vegetables that are recommended to enhance immune system.

The current study have some limitation. First, the sample is limited to adults who had access to a smart phone or computer to complete the online survey, which may have led to selection bias. Second, data were self-reported by respondents, which could lead to recall bias. Third, participant's income was assessed only during the pandemic and its information before the Corona out-break was not collected, which may make the results of the association between socioeconomic status and food intake before and after Corona unreliable. Despite these potential limitations, these findings provide valuable insights into how the COVID-19 out-break has impacted adults' dietary food intake, body weight, and dietary supplements intake.

In conclusion, our findings revealed that there were significant changes in body weight, dietary habits and supplement intake during COVID-19 pandemic among Iranian population. In addition, before and during COVID-19 pandemic weekly dietary intakes were associated with socioeconomic status. The information from the present study would be useful for health professionals and policymakers.

## Data availability statement

The original contributions presented in the study are included in the article/Supplementary material, further inquiries can be directed to the corresponding author.

## Ethics statement

The studies involving human participants were reviewed and approved by the Research Ethics Committee of Shiraz University of Medical Sciences (IR. SUMS.REC.1401.344). The patients/participants provided their written informed consent to participate in this study.

## Author contributions

MM and SFS contributed to the study's design and data collection. SPM contributed to the data analysis. SPM, SFS, and MM wrote the manuscript. All authors approved the final version of the manuscript.
