# Supplementary material for: Changes in dietary habits and weight status during the COVID-19 pandemic and its association with socioeconomic status among Iranians adults
Source: Front Public Health. 2023 Jan 12;10:1080589. doi: 10.3389/fpubh.2022.1080589 (PMC9877516; doi:10.3389/fpubh.2022.1080589)
Supplement: Supplementary file 1 [file Table_1.DOCX]

**Research Questionnaire**

**Part 1: Demographic characteristics**

1. Sure name, Family name:
2. Phone Number:
3. Sex:
4. Age: ………… years old
5. Educational level: □ <12 years □ 12 years □ 12-1 years □ >16 years
6. Monthly income level(million Rls): □ Less than 30 □ 30-60 □ 60-90 □ Above 90
7. Marital status: □ Single □ Married □Widowed or Divorced
8. Do you smoke? Yes □ No□
9. Weight: ………. Kg height:……….cm

**Part 2: health status**

1. Do you previously infected with COVID-19? Yes □ No□
2. Do you have any disease? Yes  No

If yes, Name it:…….

**Part 3: Dietary intake and physical activity:**

The questions below ask you about your dietary intake; please write the amount of the foods consumed

| **Food items** | **Before covid-19 pandemic** | **During covid-19 pandemic** |
| --- | --- | --- |
| **Whole bread (serving/week)** |  |  |
| **Legumes and beans (food spoon/week)** |  |  |
| **Soy bean (food spoon/week)** |  |  |
| **Nuts (number/week)** |  |  |
| **Seeds (food spoon/week)** |  |  |
| **Milk (serving/week)** |  |  |
| **Yogurt (serving/week)** |  |  |
| **Cheese (serving/week)** |  |  |
| **Red meat (serving/week)** |  |  |
| **Poultry (serving/week)** |  |  |
| **Fish (serving/week)** |  |  |
| **Canned fish (serving/week)** |  |  |
| **Egg (number/week)** |  |  |
| **Homemade fast foods (serving/month)** |  |  |
| **Take out fast foods (serving/month)** |  |  |
| **High Vitamin C vegetables (** **sweet pepper,** **Broccoli and other cruciferous vegetables,….) (serving/week)** |  |  |
| **High Vitamin C fruits (**  **oranges, kiw,** **Strawberries,…) (serving /week)** |  |  |
| **Green, yellow fruits and vegetables (serving/week)** |  |  |
| **Onion/garlic (serving/week)** |  |  |
| **Dried fruits (number/week)** |  |  |
| **Natural fruit juices (glass/week)** |  |  |
| **Commercial fruit juices (glass/week)** |  |  |
| **Carbonated drinks (glass/week)** |  |  |
| **Water (glass/day)** |  |  |

**Physical activity**

1. During the last 7 days, on how many days did you do vigorous physical activities like heavy lifting, digging, heavy construction, or climbing up stairs as part of your work?

Think about only those physical activities that you did for at least 10 minutes at a time

_____ days per week

□ No vigorous job-related physical activity Skip to question3

1. How much time did you usually spend on one of those days doing vigorous physical activities as part of your work?

_____ hours per day

_____ minutes per day

1. Again, think about only those physical activities that you did for at least 10 minutes at a time. During the last 7 days, on how many days did you do moderate physical activities like carrying light loads as part of your work? Please do not include walking.

_____ days per week

□ No moderate job-related physical activity Skip to question 5

1. How much time did you usually spend on one of those days doing moderate physical activities as part of your work?

_____ hours per day

_____ minutes per day

1. During the last 7 days, on how many days did you walk for at least 10 minutes at a time as part of your work? Please do not count any walking you did to travel to or from work.

_____ days per week

□ No job-related walking Skip to question 5

1. How much time did you usually spend on one of those days walking as part of your work?

_____ hours per day

_____ minutes per day

The last questions are about the time you spend sitting while at work, at home, while doing course work and during leisure time. This may include time spent sitting at a desk, visiting friends, reading or sitting or lying down to watch television. Do not include any time spent sitting in a motor vehicle that you have already told me about.

1. During the last 7 days, how much time did you usually spend sitting on a weekday?

_____ hours per day

_____ minutes per day

1. During the last 7 days, how much time did you usually spend sitting on a weekend day?

_____ hours per day

_____ minutes per day

**Part4: Nutritional supplement consumption:**

1. Did you use vitamin D supplement before covid-19 pandemic? Yes □ No□
2. Do you use vitamin D supplement before covid-19 pandemic? Yes □ No□
3. The questions below ask you about your Nutritional supplement; Please write the amount of the supplement you used.

| **Vitamin C (number/week)** | **Before covid-19 pandemic** | **During covid-19 pandemic** |
| --- | --- | --- |
| **Zinc (number/month)** |  |  |
| **Calcium (number/week)** |  |  |
| **Calcium + Vitamin D (number/month)** |  |  |
| **Multivitamin (number/month)** |  |  |
| **Vitamin C (number/week)** |  |  |
